# Barriers and recommendations for harm reduction services among people living with HIV in Manitoba, Canada: a qualitative study

**DOI:** 10.3389/fpubh.2025.1636409

**Published:** 2025-09-22

**Authors:** Enrique Villacis-Alvarez, Katharina Maier, Margaret Haworth-Brockman, Heather Pashe, Joel Baliddawa, Nikki Daniels, Rebecca Murdock, Robert Russell, Clara Dan, Freda Woodhouse, Susie Cusson, Marj Schenkels, Ken Kasper, Lauren J. MacKenzie, Laurie Ireland, Kimberly Templeton, Yoav Keynan, Zulma Vanessa Rueda

**Affiliations:** ^1^Department of Medical Microbiology and Infectious Diseases, Rady Faculty of Health Sciences, University of Manitoba, Winnipeg, MB, Canada; ^2^Criminal Justice, The University of Winnipeg, Winnipeg, MB, Canada; ^3^Department of Community Health Sciences, Rady Faculty of Health Sciences, University of Manitoba, Winnipeg, MB, Canada; ^4^National Collaborating Centre for Infectious Diseases, Rady Faculty of Health Sciences, University of Manitoba, Winnipeg, MB, Canada; ^5^Peer Research Team, Alltogether4IDEAS, Winnipeg, MB, Canada; ^6^The Manitoba HIV Program, Winnipeg, MB, Canada; ^7^Department of Internal Medicine. Rady Faculty of Health Sciences, University of Manitoba, Winnipeg, MB, Canada; ^8^Health Sciences Centre, Shared Health, Winnipeg, MB, Canada; ^9^Nine Circles Community Health Centre, Winnipeg, MB, Canada; ^10^Department of Family Medicine, Rady Faculty of Health Sciences, University of Manitoba, Winnipeg, MB, Canada; ^11^Faculty of Medicine, School of Health Sciences, Universidad Pontificia Bolivariana, Medellin, Colombia

**Keywords:** HIV, substance use, harm reduction, health-related harms, qualitative research, barriers, Manitoba

## Abstract

**Introduction:**

In 2022, the province of Manitoba, Canada, recorded its highest increases in substance-related deaths and new HIV diagnoses. The COVID-19 pandemic exacerbated access barriers to harm reduction services across the country. Given the intertwined relationship between HIV and injection substance use, we sought to better understand People Living with HIV’s (PLHIV) access barriers to harm reduction services, and recommendations for improved care.

**Methods:**

This qualitative study was co-designed by and co-executed with people with lived experiences in HIV and substance use. The data collection process encompassed a semi-structured in-depth qualitative interview with PLHIV followed by three quantitative questionnaires and was conducted between October 2022 and May 2023 in HIV clinics. Descriptive statistics were performed to illustrate substance use practices, and we employed reflexive thematic analysis to generate themes and explain shared patterns of meaning across participants in relation to our research question.

**Results:**

We developed two themes to explain the qualitative data: (1) *knowledge about and availability of harm reduction services*, and (2) *safer substance use and supervised consumption sites*. In the first theme, participants described being aware of the different harm reduction services in their community, but recounted several access barriers limiting service uptake, including restrictive service times and limited mobile services. These limitations increased participants’ likelihood of sharing injecting equipment, and produced stress and anxiety about lacking access to safe supplies. In the second theme, participants discussed what they consider “safe” spaces for using substances, highlighting the importance of autonomy and privacy where they can use without fear of stigma and interference. Thus, to make substance use safer in Manitoba, participants advocated for the implementation of supervised consumption sites to ensure the availability of non-judgmental spaces where they can find and use safe injecting supplies, trained staff, and connections to health and social supports.

**Discussion:**

PLHIV who use substances face many hurdles when seeking harm reduction and health services. It is essential to implement new strategies centred on the lives of PLHIV who use substances to address the unprecedented rates of HIV diagnoses, health-related harms, and substance-related deaths.

## 1 Introduction

The province of Manitoba, Canada, has struggled to address the effects of a toxic drug supply, reporting 467 and 445 substance-related deaths^1^ in 2022 and 2023, respectively (1). While substance use and overdoses affect people across Manitoba, the highest reported substance-related incidents (including overdoses) occur in the province’s largest city, Winnipeg (2). In parallel, Manitoba has also struggled to address rising HIV rates, with injection use (56.2%) being the second most common self-reported mode of HIV acquisition during the province’s highest increase in HIV diagnoses (2018–2021) (3–5). Recent findings suggest a disease cluster of increased HIV diagnoses, sexually transmitted and blood borne infections (STBBIs) and mental health conditions affecting historically oppressed and underserved communities including people who inject substances and those experiencing houselessness (4, 5).

Since the onset of the COVID-19 pandemic, people who use substances have reported increased consumption while having challenges accessing harm reduction programs across Canada (6, 7). Harm reduction refers to a philosophical and practical recognition that strategies must be in place to minimize the personal, health, social, and economic effects of substance use (8). Ideally, harm reduction provides context-based practical interventions to minimize the risks associated with substance use (8). Programs distributing safe injection equipment have proven to be safe and cost effective in preventing HIV and other infections, as well as overdoses (9). While these programs are common examples of harm reduction services, there are additional wraparound programs that include opioid agonist therapies, counselling, clean and safe consumption rooms, withdrawal management (detox), addiction treatments, and rehabilitation programs, substance testing, among others (8).

Despite the evidence supporting harm reduction services, recent survey data from people seeking harm reduction supplies in Winnipeg found barriers such as inaccessible service times, lack of transportation options and phones to call mobile services (10). Recommendations to increase safety and access to services included expanding Indigenous-led programs, affordable housing, and supervised consumption sites (10). Qualitative findings from our research team found that People Living with HIV (PLHIV) who also use substances faced increased barriers (e.g., discrimination, inflexible appointment times) when seeking and accessing HIV care (11). The social and health interactions between HIV and increased substance use can result in poor health outcomes for PLHIV (12–14). In particular, methamphetamine use, which was the substance of choice (93.4%) among those newly diagnosed with HIV in Manitoba between 2018 and 2021, has been associated with higher HIV viral replication, inflammation, and immune activation (15–18). Likewise, reusing and sharing of needles and associated equipment increases the transmission of HIV, Hepatitis B and C, and other bacterial and fungal infections (9, 19, 20). Harm reduction services play a key role in ameliorating these negative health outcomes.

The interaction between the disease clusters mentioned above indicates the presence of a syndemic—multiple health conditions closely interacting with each other which are produced, reproduced, and compounded by context-specific social factors creating negative health and social outcomes among underserved populations (12, 21, 22). The objective of this study was to understand barriers and recommendations to improve harm reduction services among PLHIV in Manitoba given how around half of new HIV diagnoses reported injectable substance use. Describing the challenges in accessing and new avenues for wraparound harm reduction services from the perspective of the people with lived experiences could inform stronger system responses to the rising HIV diagnoses and substance-related deaths.

## 2 Methods

This study is situated within a larger collaborative multidisciplinary project to produce transformational research and advocacy related to HIV and STBBIs across Manitoba. A full protocol on theoretical, community involvement, and methodological procedures underpinning this research, including quantitative and qualitative data analyses, has been published elsewhere (23). This study was approved by the University of Manitoba Health Ethics Research Board (HS25572; H2022:218), the First Nations Health and Social Secretariat of Manitoba, Nine Circles Community Health Centre, Shared Health Manitoba (SH2022:194) and 7th Street Health Access Centre.

### 2.1 Setting

This study was conducted in Manitoba, located in central Canada, with a population of 1.4 million people (24). Most of the population resides in Winnipeg (~800,000) and Brandon (~55,000), and the remaining population lives in rural and remote communities. At the time of data collection, Manitoba did not have a federally approved safe consumption site. Data collection took place at the three sites of the centralized Manitoba HIV Program located in Winnipeg and Brandon.

### 2.2 Study participants

This study was co-designed, co-executed, and co-authored from its inception with the guidance and active involvement of people with lived experiences of substance use, HIV, and socio-economic marginalization (23). Individuals with a medically confirmed HIV diagnosis, who were at least 18 years old, and resided in Manitoba were eligible to participate in the study. Purposive sampling was used to include a diverse sample of men, women, and non-binary persons, from different race/ethnic backgrounds, and people with diverse substance use experiences. Community-based recruitment included referrals from service providers working alongside PLHIV, printed posters in community organizations, social media posters, and in-person events at the Manitoba HIV Program clinics. Recruitment continued until thematic saturation was reached.

### 2.3 Data collection

Data collection took place between October 2022 and May 2023. The field team included a research associate, peer researchers, and the Indigenous Cultural Advisor. The co-development process for the data collection materials along with the materials themselves are publicly available elsewhere (23). Data collection sessions were held in a private room at the HIV clinics. Each session started by obtaining the participant’s informed consent in writing or verbally, followed by an in-depth semi-structured interview. There was a break after the interviews and then participants completed three surveys: a Sociodemographic and Life Circumstances Questionnaire, Childhood Trauma Questionnaire (25), and Empower-Making Decisions Survey (26). All surveys were paper-based, and participants could complete them themselves or receive assistance for oral completion. Data collection sessions lasted between one and two and a half hours. Interviews were audio recorded and transcribed employing Otter.ai as it provides time saving processing for lengthy transcripts. Then, transcripts were checked for accuracy by the study’s research associate (EVA). Results from the first survey on sociodemographic characteristics and substance use practices, which was co-developed with peer researchers (23), are presented in this paper. Further, we report on participants’ qualitative narratives using substances and harm reduction services.

### 2.4 Data analysis

Descriptive statistics were performed for participant demographics and substance use practices. We employed NVivo® 12 Pro for analysis of the qualitative data following reflexive thematic analysis developed by Braun and Clarke (27). Reflexive thematic analysis enables researchers to develop patterns and themes in the data while recognizing their own subjectivity in the process (28, 29). EVA spent considerable time in the HIV clinics, conducted the interviews, and reviewed the transcripts which enabled deep engagement and familiarization with the data. The analysis started with open coding to create initial codes by EVA. Through collaborative meetings KM and ZR reviewed initial codes and discussed with EVA the clustering of codes that would inform potential themes. Once all codes were created, we consolidated them and generated two potential themes to explain common patterns across participants’ narratives. We refined those themes and wrote the report to explain our findings. We include participants’ quotes, ID numbers, and self-reported genders to contextualize our qualitative findings.

## 3 Results

Thirty-two participants completed the survey and interview. Table 1 presents participants’ sociodemographic characteristics, while Table 2 describes participants’ self-reported substance use practices. The average age of substance use onset was 15.7 years, with a range from 6 to 30 years. Marijuana (*n* = 17) and alcohol (*n* = 8) were the most common first used substances. Eleven participants (40.7%) reported “heavy substance use” (i.e., one or more times per day) when they first started. Sixteen participants described their injection practices with 10 (62.5%) indicating “all of the time” when asked if they use new needles every time. Interestingly, only seven participants (43.75%) mentioned “never” injecting with a syringe used by somebody else, and six (37.5%) selected “never” for having someone else inject their substances for them. Over half of participants (56.25%) had a safe place to inject, and most of them (87.5%) knew where to find harm reduction supplies.

**Table 1:** Participants’ sociodemographic characteristics.

Variable (*N*)	Frequency *n* (%)
Age in years (*N* = 32)	
Mean (Range)	44.03 years (24–63)
Gender Identity (*N* = 32)	
Woman	10 (31.3)
Man	18 (56.3)
Non-Binary	1 (3.1)
Two-Spirit	2 (6.3)
Other	1 (3.1)
Sex (*N* = 32)	
Male	21 (65.6)
Female	10 (31.3)
Prefer not to say	1 (3.1)
Sexual Orientation (*N* = 32)	
Gay	8 (25)
Bisexual	6 (18.8)
Heterosexual	15 (46.9)
Other	2 (6.3)
Prefer not to say	1 (3.1)
Cultural Background (*N* = 32)	
Indigenous—First Nations	15 (46.9)
Indigenous—Métis	4 (12.5)
White/European	4 (12.5)
Southeast Asian	4 (12.5)
Other	5 (15.6)
Income (*N* = 31)	
<10,000 CAD/Year	12 (37.5)
10,000–19,999 CAD /Year	8 (25)
20,000–29,999 CAD /Year	2 (6.3)
30,000–39,999 CAD /Year	3 (9.4)
40,000–49,999 CAD /Year	3 (9.4)
>50,000 CAD /Year	1 (3.1)
Prefer not to say	2 (6.3)

**Table 2:** Participants’ self-reported substance use practices.

Questions	Frequency (%)
Do you currently use substances? (*N* = 30)^a^	
Yes	22 (73.3)
No	8 (26.7)
How old were you when you started using substances? (*N* = 30)	
Mean (Range)	15.7 years (Range = 6–30)
What substance did you start with?^b^ (*N* = 30)	
LSD (Lysergic acid diethylamide)	1
Alcohol	8
Cannabis	17
Opioids	2
Cocaine	2
Methamphetamine	1
Tobacco	2
Substance use pattern when started (*N* = 27)	
Heavy use	11 (40.7)
Moderate use	3 (11.1)
Light use	5 (18.5)
Very light use	8 (29.6)
Substance use pattern currently (*N* = 31)	
Heavy use	6 (19.4)
Moderate use	7 (22.6)
Light use	3 (9.7)
Very light use	5 (16.1)
Not using	10 (32.3)
Injection Practices (*N* = 16)	
Use new needles every time	
All of the time	10 (62.5)
Some of the time	6 (37.5)
Not very often	0
Never	0
Inject with syringe used by somebody else	
All of the time	0
Some of the time	2 (12.5)
Not very often	7 (43.75)
Never	7 (43.75)
Bleach needles to reuse them	
All of the time	1 (6.25)
Some of the time	1 (6.25)
Not very often	2 (12.5)
Never	12 (75)
Use a spoon or water filter used by somebody else	
All of the time	1 (6.25)
Some of the time	1 (6.25)
Not very often	4 (25)
Never	10 (62.5)
Have someone else inject your substances for you	
All of the time	1 (6.25)
Some of the time	7 (43.75)
Not very often	2 (12.5)
Never	6 (37.5)
Have a safe place to use	
All of the time	9 (56.25)
Some of the time	5 (31.25)
Not very often	1 (6.25)
Never	1 (6.25)
Know where to find harm reduction supplies	
All of the time	14 (87.5)
Some of the time	2 (12.5)
Not very often	0
Never	0

a Prioritizing a trauma-informed approach to our data collection process, participants were reminded to answer questions they feel comfortable which is why some answers do not add up to the total 32 participants.

b Categories are not mutually exclusive and therefore percentages may not add up to 100.

### 3.1 Findings from interviews

Two themes were developed to explain the qualitative data: (1) knowledge about and availability of harm reduction services, and (2) safer substance use and supervised consumption sites.

#### 3.1.1 Knowledge about and availability of harm reduction services

*I didn't know what that was [harm reduction]. Somebody was telling me, where do I get supplies?’ I said, ‘Go to Nine Circles [HIV clinic], they will have supplies* (Participant 45, Woman).

All participants were able to name local organizations that distribute harm reduction supplies, with many able to recite their street addresses, phone numbers, and hours of operation. Those who accessed these organizations explained typically picking up many supplies and sharing it with other people who use substances, especially with people who cannot get them themselves because they are too “*sick*” or “*spaced out*” (Participant 57, Non-binary). Participant 57, who explained frequenting a local harm reduction organization “*on a weekly basis*” said they keept going there “*even if I wasn’t using them*” because:

*“I know a lot of people that are using it too. And so, I would just, “here” [hand motion]. Or some of the people I knew that would just be too spaced out, like too freaking high to go by [themselves], and I [would] say like ‘here you go’”* (Participant 57, Non-binary).

Participant 57 portrayed how harm reduction supplies are distributed within the community of people who use substances, emphasizing an attitude of communal care to ensure safe substance use. Although most participants knew where to acquire supplies, including those who did not routinely access these organizations or use substances, many participants emphasized several barriers to conveniently and comfortably utilizing these supports. The traditional 9–5 business hours (Monday to Friday) when services are open was one of the main access barriers recounted across narratives. Participants reflected that many people who use substances, including some of them, used substances outside these times, thus needing clean supplies outside regular business hours. While some participants were able to ‘stock up’ on their supply or rely on others to share with them (see Participant 57), for others, these fixed business hours created tangible risks, such as reusing supplies. Participant 46 recounted having to use her “*dirties*” (previously used equipment) because she was unable to get new supplies on the weekend:

*Sometimes I would have to use my dirties because I forgot to go on Friday to get my supplies [have] to go and wait until Monday. So maybe having some places open on the weekends and even like after hours, like four o’clock, five o’clock in the morning. Somewhere you can just call because people are willing to just take a cab to wherever they have to go to get cleans. For me, if I didn’t have a clean, I couldn’t ever get my shot. And sometimes when I had to use my dirties, I was going five hours straight, six hours straight trying to get my shot, and I just couldn’t get it, very frustrating* (Participant 46, Woman).

Participant 46’s emphasis on people’s willingness to attend an available after hour service suggests that if these services were available, people would reach them even with transportation barriers. Like the frustration experienced by participant 46, many other shared heightened anxiety and physical repercussions due to the lack of new harm reduction supplies. Participants were aware that sharing or reusing injecting equipment risks negative health outcomes, with some disclosing that they had contracted STBBIs and HIV from sharing equipment and stopped these practices after learning they had a disease. However, even with an awareness of the health consequences and intentions to not share or reuse injecting equipment, participants found it difficult to get new supplies, making it necessary to ‘plan ahead’—something participants described as stressful when their substance use lead to constant immediate needs:

*I remember over the weekend, I couldn’t find a clean nowhere. So, I had to stock up every Friday to make sure I had cleans for the whole weekend. That was fucking awful… Because I would be out just worrying about my next fix and not care or nothing* (Participant 41, Woman).

While participants knew of the one mobile delivery service for people who cannot go to an organization in person, they shared similar frustrations that its hours were limited to weekdays and that it was inaccessible for those without phones. Participant 55 (Woman), while praising the mobile service, noted these limitations:

*“So, we call in for like fucking boxes, right, so they bring it which is great. It’s a fucking great service. Drop off supplies, they are eventually good on the weekdays, but like weekends would be great if I could get the fucking vans going on weekends […]. Because like it fucking annoys me like a lot of them* [harm reduction services] *only go like nine to five only. Yeah, like there should be like an overnight person that’s here handing out things.”* (Participant 55, Woman).

By Sunday, participants’ individual supply of new equipment (and therefore the ‘collective’ supply in their communities) was typically depleted, heightening people’s risk of having to reuse equipment or rely on other means to try to secure new ones:

*So, the worst is Sundays. I don’t know why because everybody seems to run out of cleans on Sundays. So, I’ve even had to get a clean from digging like fucking garbage bins. I found, like. unopened packs; I’ll use that. I finally went digging through this huge pile of garbage to find a clean.*

The narratives presented above highlighted how even with the best intentions to use substances safely, multiple organizational barriers hindered this goal. While harm reduction services are in place to support the harms of substance use, many participants in this study associated these resources with the anxiety and fear of accessing them or, worse, not having them available.

#### 3.1.2 Safer substance use and supervised consumption sites

Across interviews, the notion of safety was discussed by many participants. In particular, many participants discussed ideas of what crafted a binary of “safe” vs. “unsafe” space for substance use. When participants recounted the characteristics that encompassed a safe space, they mentioned preferring to use substances in places that offered privacy, autonomy, and familiarity. These values were discussed in relation to the amount of control participant would have of their contexts:

*Privacy, privacy. And [that] you can lock the doors. You don't have to worry about the general public. It's my own business, I'm gonna do drugs and I go and hide from the world. I do drugs to do my thing. I don't bother people. So, leave me alone* (Participant 51, Man).

Participant 51’s use of phrases such as *my own business*, highlighted how a shared frustration frustrations towards some misconceptions held in the general public about what substance use entails. Many participants recounted how these misconceptions were informed by associating people using substances with becoming violent. Participants wanted people to recognize that there is more to violence in their substance use, and that many people engage in substance use as a private affair. Participant 51 highlighted the importance of control in creating a safe space by describing actions such as locking doors, not worrying about the public, and doing his own “*thing*.”

Some participants said they prefer to have a peer with them while using in the event of a negative experience (including overdose). Having a trusted person provided control and certainty over potential negative outcomes and a reassurance that someone is looking out for their safety and wellbeing.

*Having someone there, some of the very friends that you can trust* (Participant 42, Non-binary).

Participants explained that they have and do use substances in places that they consider unsafe, though they noted that this increases the risk of missed “*hits*” or “*shots*” (i.e., an injection does not reach the vein) which can result in infections. For example, using substances in public spaces where people must watch for police or people they fear may act violently toward them can interfere with using substances, as well as unsanitary places where they could acquire infections. Participants 32, 55, and 59 provided eloquent narratives highlighting the heightened fear and anxiety that comes from using substances in places perceived as unsafe:

*I feel every time I see a fucking cop cruiser they are always fucking saying something to me. (…) What they say to me is I got my eyes on you! Or they are saying like I'm watching you, like something like that is what they say to me. I'm like, what the fuck for?! Unsafe (…) scared*. (Participant 32, Man)

*Not having privacy was just like, unsafe for getting dirty. Places are fucking scary. Some bathrooms are fucking filthy and not having those clean containers [to dispose needles]* (Participant 55, Woman).

*What makes it unsafe. Well, when you're sitting around a bus shack or wherever and you're trying to hit and other drug addicts are coming around. They're hurting. That's what makes it unsafe. Because a lot of them out there are bad people. They're walking around with machetes and stuff.* (Participant 59, Man).

Across participants, places that felt unsafe for using their substances brought up negative emotions such as fear and anxiety. Participant narratives also highlighted the importance of perceiving their spaces of use as safe given the number of negative experiences and consequences that can occur when they are not. Participants 55 and 59 reflected on two different, but associated, negative experiences that could occur from using in unsafe places such as using substances in unsanitary conditions and potential physical interactions with other people.

The primary strategy participants recommended to make substance use safer was the creation of supervised consumption sites in Manitoba. All participants with previous or current experiences with injection use supported supervised consumption sites and provided extensive commentary on why they would better support their communities as they felt supervised consumption site would save people from dying due to the ongoing drug poisoning crises. Even participants who had never used injectable substances reflected on the many benefits that these places would bring. Participants were hopeful about these sites, with many speaking fervently over the potential benefits these places could bring for people across the province. Among these benefits, many participants highlighted the opportunities these sites could create to provide holistic care for people who use substances, such as mental health and social supports. For instance, participant 51 explained how people could be better supported if these sites existed:

*Safe injection sites will save lives. It is a place where you can get a pat on the back and hear that you're not alone. It takes a special person and you know when people have that support in there it stops suicide. It stops-it empowers someone to be like ‘no, maybe I don't want to do this anymore’. It's like my mom watching me, right? People feel it is a place, where they won't be judged to be themselves. I can go there, I can talk to someone, I'm not judged. And I feel like I could really get help if I need it, if I really need help. Rather than have someone shooting up in the bus shelter? It was 9 o’clock in the morning, people are going to work but I don't give a fuck because I needed to shoot up. I just wanted to get high because I was just not feeling it. Those safe injection sites, they would help so much in people going to one place where they know they can go and be safe and get some help. You know, you can come in here, no problem. It should run 24/7. It has to be 24/7* (Participant 51, Man).

It is important to note how this participant did not mention any of the medical or biological benefits these sites might also provide (e.g., treating overdoses on site) but instead focused on the psychosocial ones. The comparison of supervised consumption sites to his *‘mom watching over him’* suggests that people who use substances are looking for a welcoming and non-judgemental space where they can look for psychosocial support, and escape from the ongoing judgement experienced in their everyday lives. Participants shared feelings of *shame* when they recounted their substance use, with many experiencing discrimination because of it. Participant 51’s emphasis on having a space where people could feel welcomed to attend pointed to a common pattern across narratives of negative interactions with health and social services due to substance use. Many participants shared past experiences of discrimination when seeking primary care services, which led them to advocate for these non-judgmental spaces. In addition, participants believed supervised consumption sites would provide people who inject substances with a clean and stigma-free environment which may reduce the risk of infections and overdoses. *Having them would be a better place where people can enjoy their high without missing* [their veins], *and then getting abscesses because of the miss, and a better chance noticing an overdose before it happens, before getting too late* (Participant 42, Non-Binary). Participant 42 placed importance on the preventative benefits supervised consumption sites could provide to them by gaining access to supports before medical concerns become urgent, such as in the case of an abscess or an overdose. Participant 46 expanded on the medical benefits supervised consumption sites could provide by emphasizing how these sites may reduce negative physical implications:

*Definitely, because I've had a couple of people say they're doctors* [inject substances for someone who also uses substances] *and do me and they really messed up my arm and it's just like, oh my goodness. Sometimes I'm just scared because my whole arm would blow right up and these people don't know what they're doing. Somewhere where you can go to do that … Maybe there wouldn't be so much needles laying around everywhere* (Participant 46, Woman).

Participants also highlighted these sites could serve as a stepping stone for people who want to access medical or social services, such as housing and addiction services. They wanted these services to be available around the clock, close to densely populated areas such as downtown Winnipeg that are easily accessible by public transportation and close to the cities’ shelters and many of its social service agencies. Participant 51 reflected on the importance of the physical location of this site to better meet the needs of those who would actually use it:

*By the Statistics Canada, you look at raw data is where you look at the numbers. Where is it gonna make the most impact? Charleswood [a high-income area] probably not. Because those guys have the money and they they're doing a daddy's garage which is bigger than my house, probably the downtown core.* (Participant 51, Man)

Apart from facilities and services for safe substance consumption, participants also desired wrap-around services (e.g., psychological, housing, financial) for people who use substances to support them beyond their substance use.

*We can put all supports in there as well. They can dispose of the needles and so that they are not in your playgrounds anymore. That they are not on your front lawn anymore. You don't have to wake up [with] an eyesore when someone passes out harm reduction supplies* (Participant 51, Man).

*So instead of just knowing that they can go there just to get high. Also like to have something else to look forward to might be incentive enough for them to say, ‘hey, maybe I could quit or maybe if I do quit right here. And now I do my last shot.’* (Participant 57, Non-binary).

While participants had a clear understanding of why these sites would help those who use substances, they also remarked on the overall negative views that some members of the public hold due to misunderstandings of what supervised consumption sites provide. Participant 64 eloquently commented on the unfavorable judgement the public has towards many harm reduction programs, yet he emphasized the need to move beyond moralizing narratives and focus on addressing people’s health and wellbeing in the current Manitoban context where many people are dying.

*Yes, I know, the concept of it seems wrong. Because like you're enticing, or you're encouraging people to get high or to use drugs, but it's better because you don't have to be on the lookout for someone that's going to report you or look for someone that's gonna hate what you're doing and try to harm you or call police. It's better that there's a place where people can go and feel safe at least for that moment, and even if they need help, there may be somebody at the door that can prevent them from getting OD* [overdose]*. Or maybe if the person needs help after they got hired or something, if they want to talk to somebody after something happened. I want to talk sometimes because I feel like, ‘why do I do this?’ It’s not just the physical act of doing it in the site, it’s having someone, having the support around. At least talking [to] somebody for a minute is still better than not. You need to offer things to be assisting to them because it's hard to for us to sit down when we need help* (Participant 64, Man).

Participants like 64 broadly agreed that with the proper resources in place many people could be connected with the specific care they need to improve their wellbeing. Narratives in this theme highlighted the ways in which participants constructed the concept of safety when using substances, with many seeking more avenues to practice safer substance use.

## 4 Discussion

The findings from this study described intertwined barriers to access and potential recommendations for harm reduction services for PLHIV who use substances in Manitoba. Many participants in this study reported younger ages for when they used substances for the first-time, with marijuana and alcohol as the most common first substances. An average early age of substance initiation along with younger people being diagnosed with HIV in Manitoba should be considered a public health priority given how prolonged use of substances may affect HIV disease progression, particularly for stimulants (3, 18).

Participants also described their injection practices, with most participants knowing where to acquire harm reduction supplies and, in the interviews, participants were able to name organizations that provide harm reduction supplies and programming. Overall, participants in this study had clear knowledge of the available harm reduction services around them, and the supplies available at these sites. Quantitative results supported by qualitative narratives suggested ongoing equipment sharing when people are using injectable substances, which place them at increased risk of acquiring additional infectious diseases (20, 30). We found that people who inject substances were aware of these risks, yet getting the supplies they need to safely use substances was often challenging. For example, participants remarked on the hardships associated with getting harm reduction supplies outside standard business hours. Participants advocated for more harm reduction services that are mobile and 24/7 to minimize reuse and supply sharing. These issues are consistent with other studies (7, 31) and our findings add to the recommendations for more flexible and person-centred harm reduction services (10).

Interviews highlighted how privacy and autonomy were among the main features shaping whether a place is constructed as safe to use substances, fear of police contact or other people acting violently and unpredictably informed how people described unsafe spaces. Similarly concerning, our research along with past findings have shown that public injecting practices carry negative legal and health consequences (32–36). Participants in our study explained that injecting substances in public spaces heightens anxiety and fear of acquiring potential infections or being criminalized. It is crucial to consider the narratives that shape unsafe feelings in public settings given how previous findings report that people who inject substances in public settings are at higher risk of overdoses, arrests, and sharing equipment (32). Participants in our study provided invaluable information that should be taken into consideration for public health efforts to promote safer substance use across Manitoba. These findings further reinforce the need to have more services that feel safe for PLHIV to receive support in their substance use to prevent negative social and health outcomes.

Lastly, the main recommendation participants made to improve harm reduction services in Manitoba was to create a supervised consumption site. This recommendation builds on previous findings from Manitoba authors who advocated for supervised consumption sites to better address the needs of people who use substances (10). The evidence is robust in support of supervised consumption sites in reducing substance-related deaths, injection-related infections, and needle sharing while improving connection with treatments and healthcare providers (37–39). According to data from the Government of Manitoba, Winnipeg emergency response teams attended 3,110 overdose incidents where naloxone was administered and 5,779 emergency department overdose presentations in 2023 (2). Past research in another Prairie Canadian province emphasized how supervised consumption sites can provide cost-saving benefits to emergency services, alleviating overwhelmed emergency response teams and hospital departments (40). In 2024, the Government of Manitoba announced development of the first Indigenous-led safe consumption site for 2025 to address the needs of people who use substances. Future research should elucidate whether this new site is meeting the needs of those who use substances.

Our study has certain limitations. Our interviews were held in HIV clinics which might have missed the experiences of people living with HIV who are unaware of their HIV status or those who are not engaged in HIV care. We used purposive sampling to include participants with different experiences in and out of HIV care and with different experiences of substances use to help ensure we heard a variety of barriers people face when connecting with their care. We hope to come back to this limitation in a future project with a larger number of participants.

## 5 Conclusion

New strategies are needed as Manitoba continues to report increasing HIV diagnoses and substance-related deaths. Findings from this study provide descriptive evidence of the barriers PLHIV who use substances face when seeking harm reduction services which is creating many health and social complications for them. Participants’ recommendations provide evidence-based strategies to improve connections with services that may prevent HIV transmission and connect more people to the supports they need. Participants in our study emphasized the need for safe consumption sites in Manitoba as they could enable better substance injection practices, connections with health and social services, reduce costs associated with substance-related emergency calls, and protect people from greater harms.

## Data Availability

The datasets presented in this article are not readily available because data collection materials are publicly available elsewhere. Individual participant data contains potentially identifiable information so it will not be publicly available. Qualitative codes, categories, and quantitative data will be available upon request on a case-by-case basis. Requests to access the datasets should be directed to Zulma.Rueda@umanitoba.ca.

## Footnotes

^1^The Epidemiology and Surveillance Unit in Manitoba describes substance-related deaths as “deaths due entirely, or at least in part, to the toxic effects of one or more substances, including alcohols. A death is considered opioid related if at least one of the substances contributing to causing death is an opioid” (1).
